# Distal anastomotic new entry tears and aortic remodeling following type A dissection repair: A systematic review

**DOI:** 10.1016/j.xjon.2024.11.004

**Published:** 2024-11-17

**Authors:** Ryaan El-Andari, Nicholas M. Fialka, Abdullah Alshehri, Ali Fatehi Hassanabad, Sabin J. Bozso, Michael C. Moon

**Affiliations:** a Division of Cardiac Surgery, Department of Surgery, University of Alberta, Edmonton, Alberta, Canada; b Section of Cardiac Surgery, Libin Cardiovascular Institute of Alberta, University of Calgary, Calgary, Alberta, Canada

**Keywords:** type A aortic dissection, distal anastomotic new entry tear, dissection

**Figure:**
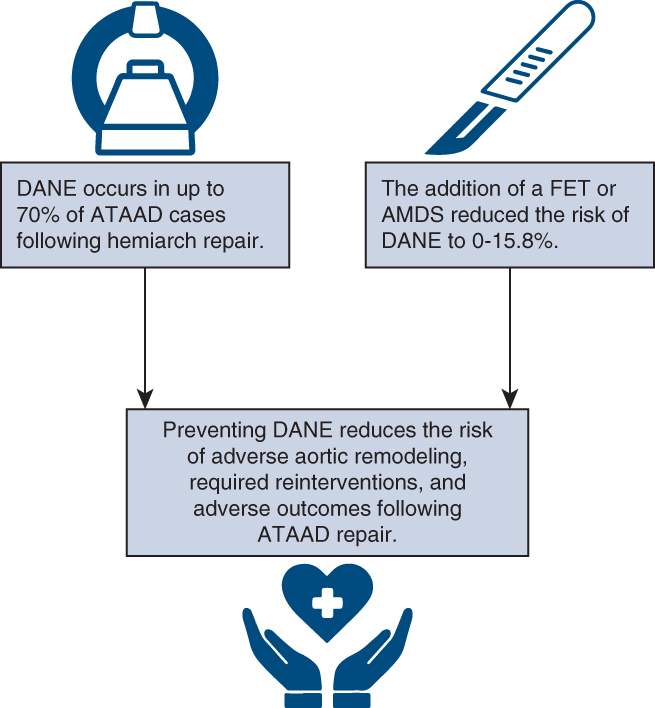
Extended arch repairs reduce the risk of DANE and adverse aortic remodeling.

**Central Message**

Rates of distal anastomotic new entry tears and adverse remodeling are reduced with more extensive repairs, such as frozen elephant trunk and AMDS hybrid prosthesis, in addition to modified distal anastomoses, such as with intimal relayering techniques.

**Perspective**

The development of distal anastomotic new entry tears (DANE) is a significant concern following acute type A aortic dissection repair, given its association with aortic expansion and adverse outcomes. More extensive repairs, such as frozen elephant trunk and AMDS, promote positive distal aortic remodeling and reduce the incidence of DANE. Strategies to mitigate DANE and promote positive aortic remodeling should be used when appropriate.

Acute type A aortic dissection (ATAAD) is an emergent, life-threatening condition.^1^ Traditionally, the focus of ATAAD, both clinically and in research settings, has been on the acute and perioperative phases. The significant surgical risk, complex management, and high rates of postoperative morbidity have garnered the bulk of the attention for these patients. Our understanding of the pathophysiology and surgical management of ATAAD have evolved over the preceding decades, with a greater emphasis on long-term outcomes and aortic remodeling.^2^, ^3^, ^4^, ^5^, ^6^, ^7^ Approaches for mitigating adverse remodeling have been highlighted in the most recent Society of Thoracic Surgeons and European Association of Cardio-Thoracic Surgery aortic guidelines.^8^ Poor outcomes related to adverse aortic remodeling have been identified, and factors that impact adverse aortic remodeling have been uncovered. One such factor is distal anastomotic new entry tears (DANE).

DANE refers to tears at the distal aortic anastomosis, thought to result from communications at the distal suture line, which create new tears allowing false lumen (FL) perfusion. DANE has been reported in up to 70% of ATAAD cases and is associated with adverse aortic remodeling and outcomes.^3^^,^^9^, ^10^, ^11^ Commonly identified following ATAAD on appropriate postoperative imaging, numerous approaches have been undertaken in an attempt to prevent DANE and in turn facilitate improved aortic remodeling and outcomes. Here we report a systematic review of the literature focused on the development and prevention of DANE and adverse aortic remodeling following ATAAD repair.

## Methods

### Data Sources

PubMed and Embase databases were searched by 2 authors. The search was conducted based on the PRISMA guidelines.^12^ The following search terms were used in the study: “aortic dissection, type A aortic dissection, distal anastomotic new entry tear, DANE, total arch replacement, hemiarch replacement, partial arch replacement (PAR), FET, frozen elephant trunk, AMDS, Ascyrus medical dissection stent, thoracic endovascular aortic repair, TEVAR”. These terms were used individually or in combination for the search. The review included any studies of ATAAD patients that reported rates of DANE, aortic remodeling, and surgical strategies to mitigate DANE and adverse aortic remodeling. Studies published from 2000 until May 1, 2024, were included in this review. The primary outcomes were the presence of DANE and measures of aortic remodeling following ATAAD repair. DANE is defined as an FL communication at the distal anastomosis on a postoperative computed tomography (CT) scan. Secondary outcomes included rates of morbidity, mortality, and reintervention, defined as any procedure on the aorta or its major branches following the initial repair.

### Study Selection

The study types included in this review were randomized control trials, retrospective study, or prospective studies. Exclusion criteria for this review included case reports, animal trials, published abstracts without an associated full text, or any study that did not report approaches to addressing DANE, rates of DANE, or aortic remodeling following ATAAD repair. Any disagreements during the literature search were resolved by discussion between the 2 reviewing authors conducting the search until consensus was reached. This review was not registered prior to collection of data.

## Results

### Overview of Included Studies

**Figure 1:**
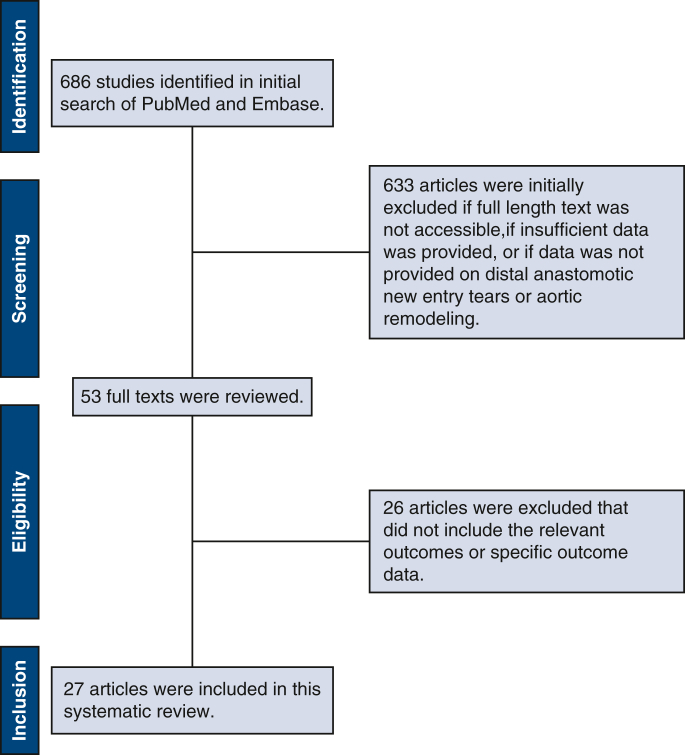
Flow diagram based on the PRISMA guidelines.

A total of 686 studies were screened, of which 27 (5 prospective and 22 retrospective studies^3^, ^4^, ^5^^,^^7^^,^^9^^,^^11^^,^^13^, ^14^, ^15^, ^16^, ^17^, ^18^, ^19^, ^20^, ^21^, ^22^, ^23^, ^24^, ^25^, ^26^, ^27^, ^28^, ^29^, ^30^, ^31^, ^32^, ^33^) were included in the final analysis (Figure 1). These studies included a total of 4035 patients with ATAAD, of whom 1120 underwent total arch replacement (TAR), 328 underwent TAR with frozen elephant trunk (FET), and 105 underwent aortic repair with an AMDS hybrid prosthesis (Artivion) implantation. Table 1 provides the characteristics of each study. Online Data Supplement provides a summary of distal anastomotic new entry tears and aortic remodeling. Table 2 reviews the morbidity and mortality rates.

**Table 1:** Characteristics of the studies included in this review

First author, year	Study type	Number of patients	Years of data collection	Follow-up period	Study population	Surgical approach/repair technique
Bozso, 2019^14^	Prospective	Total: 16	2017-2018	130 d	Acute DeBakey I aortic dissection or intramural hematoma	Surgical aortic repair with AMDS implantation
Bozso, 2021^13^	Prospective	Total: 47	2017-2019	631 d	Acute DeBakey I aortic dissection	Surgical aortic repair with AMDS implantation
Bozso, 2024^15^	Prospective	Total: 47	2017-2019	3 y	Acute DeBakey I aortic dissection	Surgical aortic repair with AMDS implantation
Desai, 2018^16^	Prospective	Total: 5		6 mo	Acute DeBakey I aortic dissections	Combined zone 2 partial arch replacement and placement of a zone 2 single subclavian branch endograft
Fukunaga, 2024^30^	Retrospective	Group 1: 74 non-TAR group Group 2: 24 TAR group Total: 98	2005-2020	4.8 ± 3.2 y	Acute DeBakey I aortic dissections	Ascending aorta and partial arch replacement with entry resection (non-TAR group) and underwent TAR with entry resection (TAR group)
Furutachi, 2019^33^	Retrospective	ET group: 30 FET group: 20 Total: 50	2010-2018	13 (7-18) 11 (9-13)	ATAAD	TAR for patients with AADA ET group: elephant trunk procedure FET group: frozen elephant trunk procedure
Iida, 2019^5^	Retrospective	Total:30	2015-2018		ATAAD	TAR with FET
Iino, 2022^18^	Retrospective	Total: 50	2016-2020	21 mo	ATAAD	TAR with FET, deploying the J graft FROZENIX into zone 1 or 2
Ikeno, 2019^7^	Retrospective	TAR: 147 Non-TAR: 120 Total: 247	1999-2016	6.5 y	Acute DeBakey I aortic dissections	TAR with ET vs non-total arch replacement
Immohr, 2023^19^	Retrospective	AMDS: 9 Control: 111	2010-2020	In-hospital	ATAAD	A total of 120 patients with combined aortic root surgery and replacement of the proximal aortic arch without further treatment of the arch beyond the innominate artery and the supra-aortic vessels were identified and included.
Inoue, 2018^21^	Retrospective	Total: 139	1997-2017	41 mo	ATAAD	TAR with the non-FET technique
Kanj, 2023^22^	Retrospective	Total: 4	2021-2022	-	Acute DeBakey I aortic dissections	David procedure and implantation of AMDS
Katayama, 2014^23^	Retrospective	Total: 120	1997-2012	104.6 mo	ATAAD	TAR with FET
Larsen, 2017^17^	Combined retrospective + prospective	Group A: 907 ascending aortic or hemiarch replacement Group B: 334 extended arch replacement Total: 1241	1996-2015		ATAAD	Ascending aortic or hemiarch replacement vs extended arch replacement. Teflon felt reinforcement 87.3%, surgical glue 60.9%
Leone, 2020^24^	Retrospective	TAA: 135 CAD: 182 Residual type A: 112 CAD: 33 Chronic type B dissections: 37 AAD: 120 Total: 437	2007-2017	2.6 y	TAA, CAD & AAD	TAR with FET
Luehr, 2023^25^	Retrospective	Total: 57	2020-2022	4.3 mo	ATAAD	AMDS implantation
Mehdiani, 2022^20^	Retrospective	AMDS: 28 Total: 97	2019-2020	30 d	ATAAD	Hybrid approach, surgical aortic repair with distal anastomosis line beyond aortic arch zone zero
Montagner, 2022^26^	Retrospective	Total: 100	2018-2021	30 d	Acute DeBakey I aortic dissections	Surgical repair of DeBakey I AAD with implantation of a noncovered stent in the arch and descending aorta.
Narita, 2023^27^	Retrospective	Proximal repair: 58 Extended arch repair: 34 Total: 92	2014-2020	31.1 mo 35 mo	Acute DeBakey I aortic dissections	Underwent proximal repair, including aortic root and/or hemiarch replacement or extended repair, including partial and total arch replacement.
Neri, 2018^28^	Retrospective	Relayering: 11	2016-2018	443 d	ATAAD	Internal reinforcement or relayering
Pan, 2017^29^	Retrospective	TEVAR for DANE: 23 TEVAR for residual intimal tear: 21	2003-2013	2.8 y	ATAAD	Reintervention TEVAR for residual or new entry tears
Rylski, 2021^3^	Prospective	Total: 86	2001-2015	31 mo	ATAAD extending at least into the aortic arch	Replacement of the ascending aorta or the ascending aorta and hemiarch replacement
Shi, 2014^4^	Retrospective	Ascending, hemiarch and ET: 71 Ascending + total arch: 84 Total: 155	2006-2011	42.7 mo 50.4 mo	Acute DeBakey I aortic dissections	Ascending, hemiarch replacement and stented ET or ascending and total arch
Takagi, 2024^32^	Retrospective	AAR, including hemiarch: 95 TAR with FET: 48	2007-2021	4.0 y	ATAAD	Ascending aortic replacement including hemiarch or TAR with FET
Tamura, 2017^9^	Retrospective	Total: 122	2007-2012	57 mo	ATAAD	Surgical repair of ATAAD
White, 2024^11^	Retrospective	Isolated hemiarch: 77 Hemiarch with AMDS: 37 Total: 114 patients	2017-2021	157 d 159 d	Acute DeBakey I aortic dissections	Either isolated hemiarch repair or hemiarch with AMDS
Yoshitake, 2020^31^	Retrospective	FET: 139 No FET: 287 Total: 426	2007-2018	46.0 mo	ATAAD	TAR with FET

*TAR*, Total arch replacement; *ET*, elephant trunk; *FET*, frozen elephant trunk; *ATAAD*, acute type A aortic dissection; *TAA*, thoracic aortic aneurysm; *CAD*, chronic aortic dissection; *AAD*, acute aortic dissection; *TEVAR*, thoracic endovascular aortic repair; *DANE*, distal anastomotic new entry tears.

**Table 2:** Morbidity and mortality rates for patients with DANE following aortic dissection repair

First author, year	Group	In-hospital, n (%)	30-d mortality, n (%)	1-y mortality, n (%)	Mortality at longest follow-up, n (%)	Stroke, n (%)	Myocardial infarction, n (%)	Acute kidney injury, n (%)	Spinal cord ischemia/injury, n (%)	Bleeding (RTOR or blood transfusion), n (%)	Other, n (%)
Bozso, 2019^14^	Total: 12		1 (6.3)			1 (6.3)		8 (50)	0		Device-related reintervention: 0 Disease-related reintervention: 1 (6.3)
Bozso, 2021^13^	Total: 46		6 (13)	9 (19.6)		3 (6.5)		5 (10.9)	0		dSINE: 0 Malperfusion-related reintervention: 3 (6.5)
Bozso, 2024^15^	Total: 47		6 (13)		3 y: 10 (21.7)	3 y: 8 (17.4)		6 (13)	0		dSINE: 0 Disease-related reintervention: 6 (13)
Desai, 2018^16^		0				0		New dialysis: 0	0		
Fukunaga, 2024^30^	Non-TAR: 74 TAR: 24	4 (5.4) 3 (12.5) *P* = .241	4 (5.4) 1 (4.2) *P* = .811	6 (8.6) 2 (9.5) *P* = .893	5 y: (12.4) (4.9) 8 y: (10.8) (10.8)	Temporary: 9 (12.2) 5 (20.8) *P* = .292 Permanent: 8 (10.8) 7 (29.2) *P* = **.030**		Hemodialysis: 1 (1.4) 4 (16.7) *P* = **.003**	TAR: 1	2 (2.7) 0 (0) *P* = .416	Aorta-related death: 0 (0) 2 (9.5) *P* = **.009**
Furutachi 2019^33^	ET group: 30 FET group: 20		3 (10) 1 (5) *P* = .64			2 (6.7) 0			2 (6.7) 0		Aortic event: 6 (22.2) 3 (15.8) *P* = .72
Iida, 2019^5^		0				1			Paraplegia: 1 Paraparesis: 1		
Iino, 2022^18^		2 (4)		(12.1)	3 y: (15.9)	7 (14)		Permanent dialysis: 2 (4)	0		dSINE: 0
Ikeno, 2019^7^	TAR: 147 Non-TAR: 120				5 y: (88.8) (84.1) 10 y: (77.2) (70.8) *P* = .207	1 (0.9) 3 (2.5) *P* = **.019**			3 (1.9) 2 (1.6) *P* = .547		TAR: 4 late aortic-related deaths
Immohr, 2023^19^	Concomitant AMDS: 9 Control: 111	1 (11.1) 24 (21.8) *P* = .68				2 (22.2) 18 (17.0) *P* = .65		1 (11.1) 24 (22.4) *P* = .68		2 (66.7) 23 (82.1)	
Inoue, 2018^21^				(3)	3 y: (5) 5 y: (5) 10 y: (7)	11 (8)	4 (3)	Hemodialysis: 3 (2)	0		
Kanj, 2023^22^			0			1 (25)		2 (50)			Device-related reintervention: 0
Katayama, 2015^23^		7 (6)	5		10 y: (23.9) 15 y: (54.9)	4 (3)	-	4 (3)	2 (2)	4 (3)	
Larsen, 2017^17^	Group A Group B	Overall: (14.2) A: (13.1) B: (17.1)			5 y: (69.4) (73.1) *P* = .83	11 (9.2) 6 (10.5) *P* = .788	28 (4.9) 9 (3.8) *P* = .476	159 (18.4) 79 (24.4) *P* = **.022**	3 (0.4) 2 (0.6) *P* = .617	All: 14/176 (8.0) 6 (5.0) 8 (14.0) *P* = **.039**	
Leone, 2020^24^	TAA AAD CAD	Overall: 65 (14.9) TAA: 26 (19.3) AAD: 21 (17.5) CAD: 18 (9.9) *P* = **.043**		Overall: (8.9)	5 y: (31.4)	Overall PND: 47 (10.8) TAA: 13 (9.7) AAD: 19 (16) CAD: 15 (8.2) *P* = .095		Dialysis: 26 (19.3) 20 (16.7) 30 (16.5) *P* = .788	8 (6) 7 (6) 9 (5) *P* = .914	12 (9) 25 (20.8) 25 (13.7) *P* = **.024**	Aortic reoperation: 6 3 16
Luehr, 2023^25^		9 (16)				2 (4)		21 (37)	0	8 (14)	AMDS collapse 5 (8)
Mehdiani, 2022^20^		1 (12.5)	1 (12.5)			0		3 (37.5)	0	1 (12.5)	
Montagner, 2022^26^		18 (18)				18 (18)		14 (14)		Hemorrhagic shock: 3 (17)	Reintervention for persistent malperfusion: 13 (13)
Narita, 2023^27^	Proximal repair Extended arch repair	Overall: 12 (13) 7 (12.1) 5 (14.7) *P* = .475	Overall: 11 (12.0) 6 (10.3) 5 (14.7) *P* = .379		Late death Overall: 26 (28.3) 18 (31.0) 8 (23.5) *P* = .440	Overall: 5 (5.4) 2 (3.4) 3 (8.8) *P* = .261		Overall, 16 (17.4) 10 (17.2) 6 (17.6) *P* = .960	Overall: 2 (2.2) 1 (1.7) 1 (2.9) *P* = .605	Overall: 7 (7.6) 4 (6.9) 3 (8.8) *P* = .515	Reintervention overall: 11 (12.0) 4 (6.9) 7 (20.6) *P* = .055
Neri, 2018^28^		1		0	0	0	3 (27)	1	0		
Pan, 2017^29^		0		1	3 y: (4.3)	0		0	0		Endoleak: 0
Shi, 2014^4^	Ascending, hemiarch, and ET Ascending and total arch	3 (4.2) 5 (5.9) *P* = .91			Aortic-related death: 0 1	TND Grade 1: 8 14 Grade 2: 3 9 Grade 3: 1 3	PND: 0 0	4 (5.6) 7 (8.3) *P* = .51	0 0	2 (2.8) 1 (1.2) *P* = .88	
Takagi, 2024^32^	AAR, including hemiarch TAR with FET	Overall: 21 (14.7) 15 (15.8) 6 (12.5) *P* = .599		(18.1) (14.6)	3 y: (22.0) (14.6)	Overall: 22 (15.4) 16 (16.8) 6 (12.5) *P* = .497		Overall, 3 (2.1) 1 (1.1) 2 (4.2) *P* = .22	Overall, 5 (3.5) 2 (2.1) 3 (6.3) *P* = .203	Overall, 5 (3.5) 4 (4.2) 1 (2.1) *P* = .513	
Tamura, 2017^9^	Posterior FL with DANE: 19 Without DANE: 27 Thrombosed FL: 47	Overall: 10 (8)		(0) (7) (0)	5 y: (8) (17) (8)						Late reoperations on DTA: 7 (8) 4 2
White, 2024^11^	Isolated hemiarch: 77 Hemiarch with AMDS: 37	9 (11.7) 5 (13.5) *P* = .768				11 (14.3) 8 (21.6) *P* = .421			0 0	6 (7.8) 3 (8.1)	Reintervention for malperfusion: 3 (3.9) 0 (0) *P* = .550 Late reintervention: 3 (3.9) 1 (2.7) *P* = .999
Yoshitake, 2020^31^	FET: 139 No FET: 287	2 (1.4) 10 (3.5) *P* = .23	2 (1.4) 7 (2.4) *P* = .50		3 y: (11.1) (10.8)	7 (5.0) 18 (6.3) *P* = .61		10 (7.2) 26 (9.1) *P* = .52	Paraplegia: 1 (0.7) 0 (0) Paraparesis: 0 (0) 2 (0.7)		Freedom from aortic-related death: (98.9) (92.3)

Bold type indicates statistical significance (*P* < .05). *RTOR*, Return to operating room; *TAR*, total arch replacement; *ET*, elephant trunk; *FET*, frozen elephant trunk; *TAA*, thoracic aortic aneurysm; *AAD*, acute aortic dissection; *CAD*, chronic aortic dissection; *AAR*, ascending aortic replacement; *FL*, false lumen; *DANE*, distal anastomotic new entry tears; *DTA*, descending thoracic aortic.

### Distal Anastomotic New Entry Tears

DANE was reported in 0% to 70% of patients who underwent PAR, in 0% to 26.0% of those with TAR without FET, in 0% of TAR with FET patients, and 0% to 11.8% of patients with aortic repair with AMDS insertion.^3^, ^4^, ^5^^,^^9^^,^^11^^,^^13^, ^14^, ^15^^,^^17^, ^18^, ^19^, ^20^, ^21^, ^22^, ^23^, ^24^^,^^26^, ^27^, ^28^, ^29^, ^30^, ^31^, ^32^ The sole study that reported 0% DANE in PAR patients described a novel intimal relayering technique to prevent DANE.^28^ White and colleagues^11^ reported a significantly lower rate of DANE in patients who underwent AMDS implantation versus those who underwent hemiarch replacement alone (*P* = .002).

### Aortic Remodeling

Aortic remodeling noting either changes to aortic diameter or FL status were reported in the majority of studies. In patients who underwent TAR without FET, 2 studies noted increased aortic diameter at longest follow-up (3-10 years).^7^^,^^30^ FL thrombosis occurred in 47% to 57.3% of patients. There were contrasting results in terms of FL thrombosis, with Ikeno and colleagues^7^ reporting significantly greater FL thrombosis following TAR compared to PAR and Fukunaga and colleagues^30^ reporting the opposite.

In patients who underwent TAR with FET, 1 study noted stable or decreased aortic diameter and/or increased FL size,^23^ whereas another found the opposite.^31^ FL thrombosis of the ascending aorta, aortic arch, descending thoracic aortic (DTA), abdominal aorta, and at the distal stent site was noted in 76%, 92.1%, 45%, 8.6% to 48%, and 91.3% to 100% of patients, respectively. Yoshitake and colleagues^31^ reported significantly greater FL thrombosis in patients who underwent FET insertion (*P* < .01).

Patients who underwent AMDS insertion, there was variety in relative changes in aortic diameter, although most studies noted a stable or decreased FL diameter and/or increased true lumen size in zones 1 to 6 (0.5-4 years of follow-up).^11^^,^^15^^,^^20^^,^^22^ FL thrombosis in the ascending aorta, DTA, and at the distal level of the stent was noted in 60.0% to 76.0%, 52.6%, and 100% of patients. White and colleagues^11^ noted significantly greater FL thrombosis and obliteration in the aortic arch (zones 1-2; *P* = .029), distal arch (zone 3; *P* = .031), and DTA (zone 4; *P* = .044) in patients in the AMDS group versus recipients of standard hemiarch repair.

### Aortic Reintervention

Rates of aortic reintervention were 3.9% to 30.7% in patients who underwent PAR (0.5-8 years of follow-up), 5.0% to 20.6% in patients who underwent TAR without FET (4.8-8 years), 2.0% to 23.1% in recipients of TAR with FET (2.6-10 years), and 0% to 13.0% in recipients of AMDS insertion (0.5-3 years).^4^^,^^5^^,^^9^^,^^11^^,^^13^, ^14^, ^15^^,^^17^^,^^18^^,^^21^, ^22^, ^23^, ^24^^,^^27^^,^^28^^,^^30^^,^^32^^,^^33^ Significantly reduced rates of aortic reintervention in patients undergoing FET compared to no FET up to 3 years of follow-up were noted by Takagi and colleagues.^32^ Unsurprisingly, Fukunaga and colleagues^30^ noted that the rate of aortic reintervention was related to FL thrombosis, with 5-year reintervention rates of 0% and 12.7%, as well as 11.6% and 2.9% in patients with a thrombosed versus a patent FL in the DTA and abdominal aorta, respectively. Rates of mortality and morbidity are reported in the Appendix E1.

## Discussion

DANE following repair of ATAAD continues to be a significant concern associated with adverse aortic remodeling and required repeat interventions. Strategies to mitigate DANE are of considerable interest. Several notable findings were identified in this review. First, rates of DANE varied greatly across the studies and the types of surgical repair. PAR tended to have the highest rates of DANE, reaching up to 70%, whereas extended arch repairs attenuated the risk of DANE.^3^, ^4^, ^5^^,^^15^^,^^17^, ^18^, ^19^, ^20^, ^21^, ^22^, ^23^, ^24^, ^25^, ^26^, ^27^, ^28^, ^29^, ^30^, ^31^, ^32^ In extended arch repairs where DANE is found, the DANE is moved distally into the distal arch or DTA. Arch devices such as FET and AMDS have been associated with further reduced rates of DANE.^5^^,^^10^^,^^15^^,^^16^^,^^22^^,^^33^ Other techniques to address DANE, such as intimal relayering or felt reinforcement, were found to be effective in reducing the risk of DANE and the rate of reinterventions.^9^^,^^17^^,^^28^^,^^30^ Although it is important to note that although these techniques are used at many centers, explicit descriptions of intimal relayering techniques or the use of felt reinforcement were infrequent in the included studies.

While rates of DANE were reduced with endovascular devices such as FET and AMDS, this did not obviate the need for reintervention. For example, the use of AMDS and FET has been demonstrated to result in a rate of DANE as low as 0%,^4^^,^^5^^,^^13^, ^14^, ^15^, ^16^^,^^21^^,^^22^^,^^24^^,^^29^, ^30^, ^31^, ^32^ although some patients have demonstrated adverse aortic remodeling or required reintervention. Given these findings, it can be inferred that FL perfusion is occurring via a mechanism other than DANE. This is likely due to such factors as dissected branch vessels and tears in other locations resulting in retrograde FL perfusion and FL expansion. This has been suggested in other investigations of adverse aortic remodeling following AMDS implantation.^2^^,^^13^, ^14^, ^15^^,^^34^ Additionally, the rates of reintervention may have been influenced by the complexity of cases, with extended arch repairs and FET more likely to be used in complex cases already at an elevated risk for adverse remodeling. Although addressing DANE and distal communications with an extended arch repair may reduce the risk of FL perfusion and adverse remodeling, it does not completely eliminate the risk, and the indications for reintervention are heterogeneous.

These findings highlight the importance of detailed evaluation of the preoperative CT scan for ATAAD. While the CT scan is often used to guide the extent of surgical management, including the proximal and distal extent of the repair, attention must be given to the contributors to FL perfusion. The presence of reentry tears in the distal aorta or branch vessels might not always be noted on initial evaluation of the CT scan but may contribute to postoperative aortic remodeling. Although DANE may be addressed by the addition of a device such as the AMDS or FET, the surgical approach must be tailored to the patient to address other contributors to future FL expansion.

### Limitations

There are several limitations to this review. The definition of DANE used in each investigation was not explicitly noted in the Methods sections of the included studies. The populations and outcomes reported were heterogeneous, precluding data pooling and aggregate analysis. Important details related to DANE, such as the distal anastomotic technique, are infrequently reported outside of studies reporting a specific modified approach.

## Conclusions

DANE continues to be a significant concern following ATAAD repair, given its association with aortic expansion and adverse outcomes. Several techniques have been used over the years in attempts to address more distal aortic remodeling as well as to mitigate DANE. This review found reduced rates of DANE in patients with more extensive repairs such as FET and AMDS, as well as in cases where the distal anastomosis was modified to mitigate DANE, such as with intimal relayering techniques. Strategies to mitigate DANE and promote positive aortic remodeling should be tailored for each individual patient.

### Data Availability Statement

The data underlying this article are available in the main article and the Appendix E1.

## Conflict of Interest Statement

Dr Moon reported receipt of consulting fees from Artivion. All other authors reported no conflicts of interest.

The *Journal* policy requires editors and reviewers to disclose conflicts of interest and to decline handling or reviewing manuscripts for which they may have a conflict of interest. The editors and reviewers of this article have no conflicts of interest.

## Appendix E1 Mortality

In-hospital mortality was reported in all but 1 study.^8^ Rates ranged from 4.2% to 21.8% in patients who underwent partial arch replacement, from 4.2% to 14.7% in patients who underwent TAR without FET, from 0% to 14.9% in patients who underwent partial/TAR with FET, and from 0% to 18.8% in patients who underwent repair with AMDS implantation. No significant differences were noted among the studies.

Regarding mid-term mortality, 1-year mortality was reported in 7 studies^15^^,^^20^^,^^23^^,^^25^^,^^29^^,^^31^ as 8.6%, 9.5%, 8.9% to 12.1%, and 19.6% in patients who underwent partial arch replacement, TAR without FET, TAR with FET, and repair with AMDS implantation, respectively. Three-year mortality rates were reported in 5 studies.^3^^,^^17^^,^^30^, ^31^, ^32^ The 3-year outcomes of the Dissected Aorta Repair Through Stent (DARTS) trial reported 21.7% mortality. Reported 3-year mortality was 5% to 11.8% in patients who underwent TAR without FET and 11.1% to 15.9% in those who underwent TAR with FET.^3^^,^^20^^,^^23^^,^^32^

Long-term mortality rates were reported in 12 studies.^3^^,^^5^^,^^6^^,^^17^^,^^19^^,^^23^^,^^25^^,^^28^^,^^30^, ^31^, ^32^ Here, 5-year mortality ranged from 4.9% to 33.6% in patients who underwent PAR, from 11.8% to 26.9% in patients who underwent TAR without FET, and from 4.3% to 31.4% in those who underwent TAR with FET. One study noted significantly improved long-term survival in patients who underwent TAR with FET (*P* = .008).^3^ Ten-year mortality was reported in 3 studies^5^^,^^6^^,^^23^^,^^29^ and was 2%, 22.2%, and 23.9% in patients who underwent PAR, TAR without FET, and TAR with FET, respectively.

## Morbidity

Rates of stroke at the longest follow-up ranged from 6.7% to 17% in patients who underwent partial aortic arch replacement, from 8.0% to 29.2% in patients who underwent TAR without FET, from 0% to 14.0% in recipients of TAR with FET, and from 0% to 37.5% in recipients of aortic repair with AMDS implantation.^2^^,^^3^^,^^5^^,^^9^^,^^13^^,^^15^, ^16^, ^17^^,^^19^, ^20^, ^21^, ^22^, ^23^, ^24^, ^25^, ^26^, ^27^, ^28^, ^29^, ^30^, ^31^, ^32^, ^33^ Of note, only 2 studies identified significantly higher rates of neurologic dysfunction in patients undergoing TAR without FET compared to PAR (Fukunaga and colleagues,^2^ *P* = .03; Shi and colleagues,^31^ *P* = .04). Spinal cord injury occurred in 0% to 1.7%, 0% to 2.9%, 0% to 6.3%, and 0% of patients who underwent partial aortic arch replacement, TAR without FET, TAR with FET, and aortic repair with AMDS implantation, respectively.

Myocardial infarction occurred in 4.9% of patients who underwent PAR and in 3.0% to 3.8% of patients who underwent TAR without FET.^19^^,^^23^ Rates of AKI ranged from 1.4% to 22.4% in partial aortic arch replacement recipients, from 2% to 16.7% in TAR without FET recipients, from 3.0% to 30% in TAR with FET recipients, and from 0% to 50% in recipients of aortic repair with AMDS implantation. Severe bleeding occurred in 2.7% to 25.9%, 0% to 14.0%, 3.0% to 14.2%, and 8.1% to 33.3% in partial aortic arch replacement recipients, TAR without FET recipients, TAR with FET recipients, and aortic repair with AMDS implantation recipients, respectively. Larsen and colleagues^19^ reported significantly increased bleeding in patients who underwent PAR compared to those who underwent TAR without FET (*P* = .039).

## Supplementary Data

Online Data Supplement

Table E1
