# Supplementary material for: Distal anastomotic new entry tears and aortic remodeling following type A dissection repair: A systematic review
Source: JTCVS Open. 2024 Nov 17;23:89–100. doi: 10.1016/j.xjon.2024.11.004 (PMC11883700; doi:10.1016/j.xjon.2024.11.004)
Supplement: Table E1 [file mmc2.docx]

**Distal Anastomotic New Entry Tears and Aortic Remodeling Following Type A Dissection Repair: A Systematic Review**

Table 2. Summary of distal anastomotic new entry tears and aortic remodeling.

| **Study name** | **Group** | **DANE (%)** | **Change in Aortic Diameter/Area** | | | **Change in True Lumen Diameter/Area** | **Change in False Lumen Diameter/Area** | **False Lumen Thrombosis/Patency (%)** | | | | | **Aortic Reintervention (%)** | | |
| --- | --- | --- | --- | --- | --- | --- | --- | --- | --- | --- | --- | --- | --- | --- | --- |
| **Bozso 2019** | Total 16 | 0 |  | | |  |  | Complete or Partially Thrombosed  Aortic arch: 11 (91.7%) cases.  Descending thoracic aorta: 11 (91.7%) | | | | | **1** (6.3) % (1/16)  (renal artery stenting) | | |
| **Bozso 2021** | Total 47 | 0 (0) | **Aortic arch (Zone A):**  Stable or decreased in 100%  **Proximal DTA (Zone B1):**  Stable or decreased 77.2%. | | | **(Zone A):**  stable or decreased 100 % of patients at 1 years  **(Zone B1):**  stable or increased in 100% of patients at 1 year. | **(Zone A):**  stable or decreased 100% of patients**.**  **(Zone B1):**  stable or decreased 97.1% | **Zone A**:  Complete thrombosis 74.3%  Partial thrombosis 10.3%  patent 15.4%.  **Zone B1:**  Complete thrombosis 52.6%  Partial thrombosis 15.8%  Patent 31.6%. | | | | | One patient required aortic growth–related reintervention in zone B1  1/47 | | |
| **Bozso 2024** | Total 47 | 0 (0) |  | | |  |  | Complete or partial FL thrombosis  Zone 1 60%  Zone 2 68.2% | | | | | Disease-related reintervention: 6 (13)   - 2 visceral stents - 2 supra-aortic vessel stents - 2 redo Bentall procedures - 1 TEVAR. | | |
| **Desai 2018** | Total 5 | 0 (0) |  | | |  |  | FL thrombosed in all patients in the stented portion and had continued flow in the FL below the stented portion from distal re-entry tears. | | | | |  | | |
| **Fukunaga 2024** | Non-TAR  TAR  Non-TAR  TAR  Non-TAR  TAR |  | Descending Thoracic Aorta (mm)  33.1 +/- 4.9  36.3 +/- 8.3 p=0.214  Aorta at Celiac Trunk (mm)  29.2 +/- 6.0  31.0 +/- 4.6 p=0.112  Infrarenal Aorta (mm)  22.0 +/- 3.9  28.6 +/- 10.0 p=0.064 | | |  |  | **DTA level:**  Complete thrombosis:  30 (46.9)  7 (36.8) 0.440  Partially thrombosed  15 (23.4)  6 (31.6) 0.474  No thrombosed  19 (29.7)  6 (31.6) 0.906 | | **Infrarenal AA level:**  Completely thrombosed  0 (0)  0 (0)  Partially thrombosed  33 (51.6)  4 (21.1) 0.019  No thrombosed  31 (48.4)  15 (78.9) 0.019 | | | Reoperation on aortic root  1 (1.4)  2 (9.5) 0.068  Freedom from downstream aorta-related reinterventions at 5 years  87.8% ± 4.5%  64.1% ± 11.0%  Freedom from downstream aorta-related reinterventions at 5 years in DTA:  thrombosed FL 100%  Patent FL 87.3% (P = **0.017**)  Freedom from downstream aorta-related reinterventions at 5 years in abdominal aorta:  Partially thrombosed FL 89.4%  Patent FL 97.1% (P = 0.113) | | |
| **Furutachi 2019** | ET group  FET group | NA  3 (15.8)  dSINE: 3 (15.8%). |  | | |  |  | Upper Position Complete FL thrombosis  ET: 15 (55.6)  FET: 13 (68.4) P = 0.50  Lower Position Complete FL thrombosis  ET: 10 (37)  FET: 9 (47) P = 0.54 | | | | | 6 (22.2)  3 (15.8) P = 0.72  An aortic event was defined as a maximal DTA diameter >50mm or reoperation for residual DTA expansion. | | |
| **Iida 2019** | **Pattern 1:** no communications between lumina postoperatively (n=12)  **Pattern 2:** the most proximal tear in the descending aorta (n=9)  **Pattern 3:** when the most proximal tear was present below the diaphragm (n=5) | 0 | Complete AoR (n = 12)  AoR (-) and enlargement: TEVAR (n = 3)  AoR (-) and no enlargement: observation (n = 5)  AoR (-) and FET retraction: TEVAR (n = 1)  (E) AoR (+) until the most proximal tear and  no AoR distally (n = 3)  Complete AoR below the diaphragm (n = 2) | | |  |  |  | | | | | TEVAR: 3 | | |
| **Iino 2022** |  | dSINE: 0 |  | | |  |  | Rate of complete false lumen thrombosis at discharge  Tracheal Bifurcation: 32 (76%)  Thoracoabdominal Transition: 19 (45%)  Celiac Artery: 15 (36%) | | | | | 1 patient required endovascular  extension for the dilatation of the descending thoracic aorta | | |
| **Ikeno 2019** | TAR  non-TAR |  | In the non-TAR group, aortic diameter had significantly increased at all 4 levels.  Levels:  TAR: Tracheal bifurcation (P < 0.001)  Th 8: improved aortic remodeling compared to the non-TAR group (P = 0.079)  Coeliac artery: aortic remodeling was significant in the TAR group at the tracheal bifurcation (P < 0.001) | | |  |  | Distal Arch FL thrombosis  **TAR:** preop 26.7%, postop 57.3%  **Non-TAR**: preop 36.8%, postop 47.3% | | | | | **Freedom from surgery:**  97.5 ± 1.8% at 5 years and 88.1 ± 5.0% at 10 years  88.2 ± 3.4% at 5 years and 73.1 ± 6.0% at 10 years P = **0.045**  **Distal aortic events**  97.2 ± 1.6% at 5 years and 84.2 ± 6.0% at 10 years  80.7 ± 4.2% at 5 years and 65.3 ± 6.7% at 10 years P = **0.013** | | |
| **Inoue 2018** |  | 0 |  | | |  |  | Patent 17 (66)  Thrombosed 9 (34) | | | | | **Reoperation**  Open repair 16 (62)  Descending aorta 6 (24)  Thoraco-abdominal 10 (38)  TEVAR 10 (38) | | |
| **Kanj 2023** |  | 0 | Stable 4/4 (100) | | | Increase by 91% in 4/4 (100) | Aortic arch:  Decreased in 1 / 4  DTA:  Decreased in 4/4 | Aortic arch:  Obliterated in 3/4 | | | | | **0** | | |
| **Katayama 2014** |  | dSINE: 1 |  | | | No change at 1 year | Obliteration 105 / 110 (95%) | 0 patency | | | | | Additional operation 11  Bentall 2  EVAR 4  Replacement of the descending aorta 1  Abdominal aorta 4 | | |
| **Larsen 2017** | Group A  Group B |  |  | | |  |  |  | | | | | 5-year freedom from death, aortic rupture and reintervention  71.1%  76.4% P = 0.54 | | |
| **Leone 2020** | TAA  AAD  CAD |  |  | | |  |  |  | | | | | Reoperation  6  3  16 | Endovascular extension  22  6  33 |  |
| **Luehr 2023** |  | **0** |  | | |  |  |  | | | | | Central collapse of AMDS: 5 (9%) | | |
| **Mehdiani 2022** |  | **0** | at zone III  960.50 P0.9453 | | | Zone III (mm)  558. P **0.0078**  T11: 463.81 P **0.0078** | Zone III (mm)  402.38 P 0.1953  T11: 262.36 P **0.0234** |  | | | | |  | | |
| **Montagner 2022** |  | dSINE: 0  0 reintervention for DANE |  | | |  |  | Complete or partial FL thrombosis, mid-descending aorta: 67 (76%) | | | | |  | | |
| **Narita 2023** | Proximal repair  Extended arch repair | dSINE: 0 |  | | |  |  |  | | | | | PR: 4 patients (6.9%) required elective aortic reintervention for significant FL dilation, 3 underwent surgical arch replacement, and 1 underwent TEVAR  ER: 7 (20.6%) required aortic reintervention, 3 underwent TEVAR following TAR with FET. | | |
| **Neri 2018** |  | 0 |  | | |  |  | Proxima l /3 of the thoracic aorta FL was obliterated in all cases but one; in 3 cases complete exclusion of the FL was obtained after the operation. | | | | | Stent graft completion  1 | | |
| **Pan 2017** |  | **dSINE: 2** |  | | |  | Before TEVAR 37.3±12.4  6 months after TEVAR 34.6±11.3 | Around the endograft stented aortic: FL Thrombosis: 21 (91.3%)  Thoracoabdominal: FL Thrombosis 2 patients | | | | |  | | |
| **Rylski 2021** |  | **CT At discharge:**  DANE: 60 (70%)  Supra-aortic arteries in 43 (50%)  Proximal DTA (28)  Lesser curvature (12)  **Follow-up CTA for 54 patients:**  Proximal DTA 12 (22)  Lesser curvature 6 (11)  Distal aorta–graft anastomosis 5 (9) | Diameter D/year P-value  At IA: 0.7 (0.04, 1.6) **<0.001**  At LCA: 0.3 (-0.4, 1.3) 0.070  At LSA: 0.6 (-0.02, 1.5) **0.002**  20 mm distal to LSA: 1.5 (0.6, 3.9) **<0.001**  40 mm distal to LSA: 1.5 (0.4, 5.3) **<0.001**  100 mm distal to LSA: 1.1 (0.02, 3.0) **<0.001** | | | Diameter D/year P  At IA: 0.7 (0.1, 1.5) **<0.001**  At LCA: 0.3 (-0.4, 1.3) 0.070  At LSA: 0.6 (-0.02, 1.5) **0.002**    20 mm distal to LSA: 1.5 (0.6, 3.9) **<0.001**  40 mm distal to LSA: 1.5 (0.4, 5.3) **<0.001**  100 mm distal to LSA: 1.1 (0.02, 3.0) **<0.001** |  | FL thrombosis  No  Partial  Complete | No aortic growth (n = 13)  2 (16.7)  3 (25.0)  8 (66.7) | | Moderate aortic growth  (n = 18)  14 (77.8)  2 (11.11)  2 (11.11) | Accelerated aortic growth  (n = 23)  15 (65.2) **0.006**  8 (34.8) 0.708  0 **<0.001** |  | | |
| **Shi 2024** | Ascending, hemiarch & ET  Ascending + total arch | 0 residual arch dilation or intervention | Enlargement of the descending aorta (>55 mm):  4  4 | | |  |  | Complete FL thrombosis 24 months  Arch:  49 (92.5)  At stent:  52 (98.1)  59 (98.3) 1.00 | Middle DTA:  28 (52.8)  35 (58.3) 0.56  Diaphragmatic level:  24 (45.3) 26 (43.3) .84 | | | | 5-year freedom  88.8  91.3% P 0.62 | | |
| **Takagi 2024** | TAR with FET  AAR, including hemiarch | d-SINE: 2 |  | | |  |  |  | | | | | Freedom from aorta-related events  **1 Year:**  90.6%  97.6%  **3 Years:**  69.3%  87.0% (**P = 0.034**) | | |
| **Tamura 2017** | Status of the residual FL:  PFL with DANE (n = 19)  without DANE (n = 27)  thrombosed FL (n = 47) | DANE n = 19 | The mean segmental aortic growth rate per year | | |  | Thrombosed FL: all 47 patients showed no significant aortic enlargement | Thrombosed FL: 47 / 93 | | | | | 6 patients required TEVAR:  4  2 | Freedom from dissection-related of the DTA at 1 year & 5 years:  93% & 66%  93% & 83%  100% at 5 years | |
|  |  |  | Proximal DTA  0.16 ± 1.8 mm  (in all groups) | Middle DTA  0.17 ± 1.3 mm  (in all groups) | Distal DTA  0.6 ± 1.3 mm,  (in all groups) |  |  |  |  |  |  |  |  |  |  |
| **White 2024** | Isolated hemiarch  Hemiarch + AMDS | 26 (43.3)  4 (11.8) **P 0.002** |  | | |  |  |  | | | | |  | | |
| **Yoshitake 2020** | FET  No FET | dSINE: 1 | 5 patients enlarged DTA  8 patients enlarged distal arch or descending | | |  |  | FL Thrombosis  87.8%  58.6% (**P < 0.01**). | | | | | Aortic reintervention:  14  20 | | |

TL, true lumen; FL, false lumen; DTA, descending thoracic aorta; TAR, total arch repair; FET, frozen elephant trunk; AAR, ascending aortic replacement; PFL, patent false lumen; ET, elephant trunk; CT; computed tomography; CTA, CT angiogram; TEVAR, thoracic endovascular aortic repair; dSINE; distal stent induced new entry.

Bold values indicate statistical significance, p<0.05.
